# Euglycemic diabetic ketoacidosis and COVID‐19 management in a term pregnant patient; a case report

**DOI:** 10.1002/ccr3.6705

**Published:** 2022-12-05

**Authors:** Fatemeh Mohammadzade, Behnaz Khodabakhshi, Elahe Amiri, Amir Bigdeli, Fahimeh Abdollahi, Alireza Fatemi

**Affiliations:** ^1^ Metabolic Disorders Research Center Golestan University of Medical Sciences Gorgan Iran; ^2^ Infectious Diseases Research Center Golestan University of Medical Sciences Gorgan Iran; ^3^ Department of Nephrology and Hypertension, Sayyad Shirazi Hospital Golestan University of Medical Sciences Gorgan Iran; ^4^ Pulmonary Ward, Department of Internal Medicine, Sayyad Shirazi Hospital Golestan University of Medical Sciences Gorgan Iran; ^5^ Ischemic Disorder Research Center Golestan University of Medical Sciences Gorgan Iran

**Keywords:** COVID‐19, euglycemic, gestational diabetes, ketoacidosis, pregnancy

## Abstract

In this case report, we report a Covid‐19 infected female patient with gestational diabetes mellitus with primary manifestation of ketoacidosis at term pregnancy and discuss the management challenges with euglycemia and a high ketone burden.

## Abstract 2

The diagnostic and therapeutic challenges associated with euglycemic DKA and its associated complications was our case highlights. Fetal death is one of the fulcrum of treatment, early detection, and timely administration of fluids and insulin.

## 1 BACKGROUND

In December 2019, a novel coronavirus later named COVID‐19 appeared in Wuhan, China, and quickly developed into a worldwide pandemic.^1^ COVID‐19 can have significant effects on pregnancy and a pregnant woman with this infection may have symptoms such as shortness of breath, fatigue, and lethargy. Pregnant women are at higher risk for developing ketosis, and concomitant infections may lower the metabolic threshold at which ketoacidosis happen.^2^

Certain hormones production during pregnancy such as progesterone, cortisol, and prolactin can increase the risk of ketoacidosis by boosting insulin resistance.^3^ Blood glucose level less than 200 mg/dl, increased anion gap metabolic acidosis, and ketonemia are the biochemical triad for euglycemic diabetic ketoacidosis (DKA).^4^

COVID‐19 may accelerate ketosis and ketoacidosis without diabetes mellitus with ketosis separately associated with increased hospital stays and mortality in younger patients.^5^ DKA is more likely developed in pregnant woman compared to non‐pregnant women with diabetes (8.9% vs. 3.1%, respectively).^6^

Diabetic ketoacidosis in pregnancy is an infrequent but critical condition that states a significant risk to the fetus. A pregnant woman is predisposed to ketosis and ketoacidosis due to the physiological state of pregnancy, with the capability to be intensified by acute stressors such as infection.^7^

In this case report, we report a COVID‐19 infected female patient with gestational diabetes mellitus with primary manifestation of ketoacidosis at term pregnancy and discuss the management challenges with euglycemia and a high ketone burden.

## 2 CASE PRESENTATION

On November 5, 2021, a 35‐year‐old female patient with gestational diabetes mellitus Gestational 3 Para 02 Labor 02 with a gestational age of 34^+5^ weeks was admitted to our hospital with a shortness of breath class 2, myalgia, headache, tachycardia, fatigue, and productive cough that caused chest pain. The condition started in the previous week and intensified 2 days ago. She also had blood glucose level of 70 at the admission. The patient had several visits to the general practitioner and received intravenous normal saline serum before admission but did not recover. Her past medical history included tachycardia in her past pregnancy and left lobe thyroid nodule that are compatible with papillary thyroid carcinoma (PTC) through fine needle aspiration which was diagnosed at eighth week of pregnancy and according to the absence of lymphadenopathy and extrathyroidal invasion, postpartum surgery was recommended. The patient was also being treated with levothyroxine due to hypothyroidism. Continuing the history of previous diseases, she also had mild gestational diabetic mellitus (GDM) since 5th month of pregnancy and had been under treatment with 6‐unit of insulin detemir.

She had a history of tachycardia in previous pregnancy and had also been treated with propranolol for the last 5 years before discontinuing it for pregnancy. During this pregnancy, she did not have any referrals for heart problems.

## 3 INVESTIGATIONS AND TREATMENT

**FIGURE 1:**
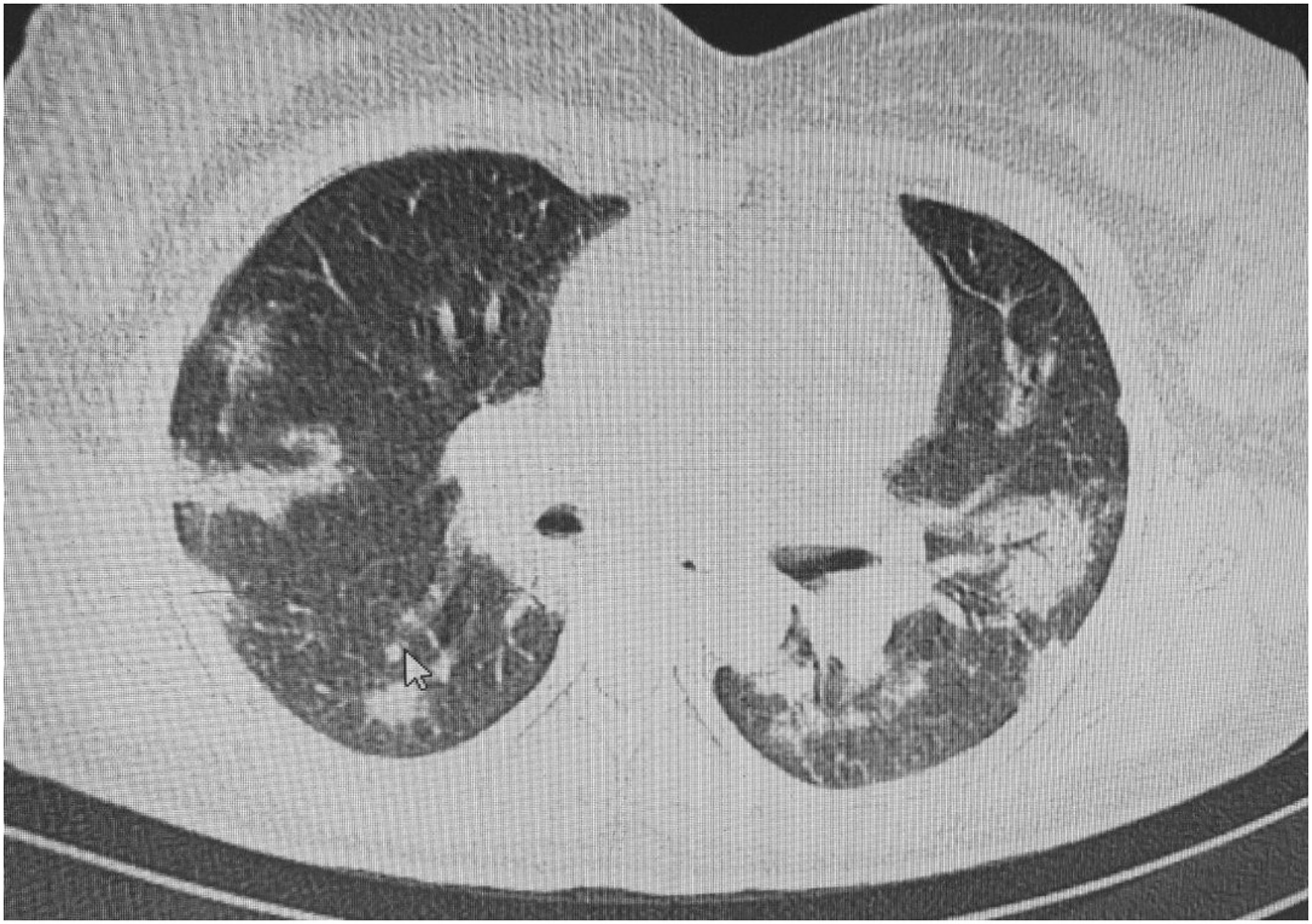
High resolution chest CT‐scan of the patient with pulmonary involvement following Covid‐19 infection showing bilateral patchy infiltration with subpleural involvement in some areas and mild grand glass opacity.

At primary presentation, the patient's shortness of breath qualified her for routine SARS‐CoV‐2 PCR testing. She had a blood glucose of 70 mg/dl, a blood pressure of 110/70 mm Hg, and a heart rate of 120 beats per minute. Her peripheral oxygen saturation was 97%, and she had a respiratory rate of 35; She also had a history of contacting her husband, who was positive for COVID‐19. The SARS‐CoV‐2 PCR test came back positive in the next day. Due to positive high‐resolution CT‐scan and pulmonary involvement of 40% and severe tachypnea, she was hospitalized at intensive care unit for 4 days. Figure.1. The patient had a platelet count of 70,000 at the admission, but no schistocytes were seen in the peripheral blood smear; therefore, thrombotic microangiopathy was ruled out. According to PH level of 7.33, PCO_2_ level of 16.7 kPa, bicarbonate level of 8.2 mmol/L, and a high anion gap of 21 mmol/L, the patient was diagnosed with ketoacidosis. Table.1.

**TABLE 1:** Relevant laboratory results at the day of admission

	Patient	Normal
Arterial blood gas		
PH	7.33	7.35–7.45
PCO_2_	16.7	4.4–6.3 kPa
Bicarbonate	8.2	23–29 mmol/L
Base excess	−9.50	−3 to 3 mmol/L
PO_2_	28.40	10–13.3 kPa
Anion gap	21	4–12 mmol/L
Venous blood sample		
WBC	11.1	04.5–11 mmol/L
RBC	3.42	4.35–5.65 mmol/L
Hemoglobin	10.2	7.5–10 mmol/L
Neutrophil	82	2.5–7.0 × 109/L
Leucocytes	10	4.0–10.5 × 109/L
Thrombocytes	70	150–400 × 109/L
Sodium	142	135–145 mmol/L
Potassium	3.6	3.5–4.5 mmol/L
ASAT	37	0–40 U/L
ALAT	27	0–34 U/L
LDH	335	0–247 U/L
CRP	+++	0–5 mg/L
ESR	84	0–29 mm/h
Creatinine	0.8	0–5 mg/L
Glucose	70	70–100 mg/dl
Ferritin	120	10–120 ng/ml
Urine		
Ketones	+++	
Glucose	Negative	Negative
Protein	Negative	Negative
Blood	Negative	Negative
Bilirubin	Negative	Negative
Nitrite	Negative	Negative
Calcium	180.4	<250 mg/24 h
Phosphorus	132.0	400–1000
PH	5	4.6–8

Abbreviations: ALAT, alanine amino transferase; ASAT, aspartate amino transferase; CRP, C reactive protein; LDH, lactate dehydrogenase; PCO2, partial pressure of carbon dioxide; PO2, partial pressure of oxygen.

A number of differential diagnoses were considered for this patient. Serum osmolarity of 296.2 mOsmol/KG, and an osmolarity gap of 7 mOsm/kg, ruled out the alcohol intoxication in this patient. High anion gap of 21 mmol/L and stage 2 chronic kidney disease epidemiology collaboration also excluded renal tubular acidosis and uremic acidosis from our differential diagnoses, respectively. D‐dimer level of 2130 ng/ml was also checked in this patient for pulmonary embolism ruling out.^8^ The patient underwent cardiac echo and had a BNP level checked for cardiomyopathy, which was reported negative. Fetal sonography was also reported to be normal during pregnancy.

**FIGURE 2:**
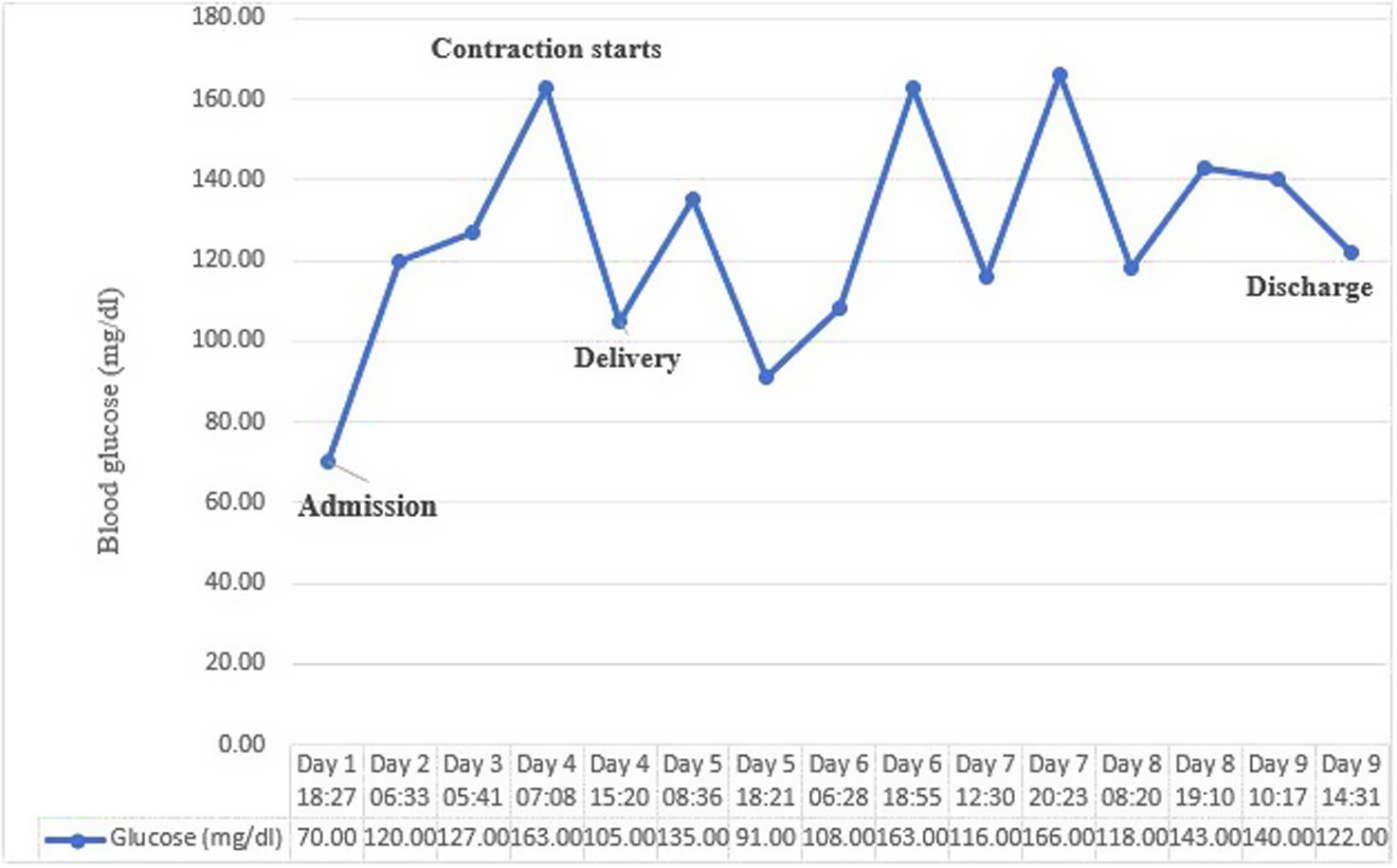
A figure showing blood sugar level variations throughout the patient's hospital stay.

Patient's blood glucose was 70 at the first presentation; therefore, serum dextrose 5% and regular insulin infusion for maintaining blood glucose level in the normal range, were established for patients' ketoacidosis. Figure.2. Extensive treatment was performed with oxygen therapy, remdesivir 100 mg, dexamethasone 8 mg, and tocilizumab 600 mg for patient's infection with COVID‐19.

Failure to correct severe acidosis of the patient in the first 4 days of hospitalization despite treatment, high respiratory rate, nonstress test (NST) disorder, closed cervix, and patients poor condition led to fetal distress and made us unable to accomplish natural vaginal delivery for this patient; Therefore, termination of pregnancy by cesarean section was performed using induction on the fourth day of hospitalization. Thyroid tests were also suitable for termination of pregnancy. By performing the mentioned workups, the patient attained a better condition.

In vital parameters, the next day were as follows: blood pressure of 115/75 mmHg, heart rate of 78 beats per minute, temperature of 37.5°C, oxygen saturation of 98%, and a respiratory rate of 22 breaths per minute. The newborn's Apgar score in the first and fifth minutes was 8 and 9, respectively. The patient's platelets count also improved and increased by 180,000 on the day of discharge.

Due to the lack of sufficient evidence for the management of these patients, based on the DKA treatment protocol in non‐pregnant patients, we performed the treatment with serum dextrose infusion 100 cc per hour, regular insulin infusion between 6 and 12 units per hour and potassium chloride administration. We also monitored the patient's blood sugar within a safe range for pregnancy.

## 4 DISCUSSION

Ketones are organized in the liver from free fatty acids. Ketosis is a result of reduction in ketone consumption which can be clinically evident by elevated blood concentrations of ketone bodies (β‐ and hydroxybutyrate, acetone acetoacetate). COVID‐19 might accelerate fat breakdown and induce ketosis, with further extension of ketoacidosis.^1^

Euglycemic DKA is a rare and acute life‐threatening metabolic emergency that the normal blood glucose may delay diagnosis and treatment. This problem is likely due to poor oral intake, treatment with insulin prior to arrival in the hospital, in pregnant women and with SGLT2 inhibitors consumption. In this case, the patient had mild GDM and was under low‐dose Levemir insulin treatment, and according to patient's self‐monitoring of blood glucose at home, the glucose levels were in the normal range for pregnancy.^9^

Diabetic ketoacidosis must be distinguished from other causes of high anion gap metabolic acidosis including lactic acidosis (which can be associated with metformin consumption), aspirin or acetaminophen toxicity, methanol, or ethylene glycol poisoning and chronic kidney disease. Although none of these disorders cause ketoacidosis, several types of acidosis may coexist, such as lactic acidosis and ketoacidosis.^10^, ^11^

In this report, we introduced a patient who has been infected with COVID‐19 virus in the context of a mild GDM and normal blood sugar with moderate pulmonary involvement and unexpectedly severe ketoacidosis. Euglycemic DKA is a rare extrapulmonary manifestation of COVID‐19 that has been reported in both diabetic and non‐diabetic patients, as well as in pregnant and non‐pregnant women.^12^

Our patient presented with shortness of breath, fatigue, and productive coughs with intensification from 2 days ago. Initially, these complaints were mainly attributed to respiratory effects of COVID‐19. Differential diagnosis was expanded when the blood gas analysis showed a high anion gap metabolic acidosis that was not due lactate or toxic agents. Urinalysis showed ketone bodies and lower limit normal blood glucose concentrations at admission. The remaining diagnosis was euglycemic DKA based on the high metabolic demand in pregnancy.

Due to the lack of proteinuria, high blood pressure, hemolysis, and increased liver enzymes, preeclampsia also ruled out in this patient.

There are several facts about the physiopathology of ketoacidosis during infection with COVID‐19. Like any other disease, these patients may not receive a proper intake due to gastrointestinal complaints which can lead to starvation or fasting DKA.^13^

On the contrary, ketogenesis increases in pregnancy and the prevalence of ketoacidosis is higher in pregnant women than in non‐pregnant women: 8.9% versus 3.1%, respectively, due to the increase in the level of hormones secreted by the placenta such as placental lactogen and prolactin, which cause insulin resistance by the antagonistic effects of insulin, and by stimulating lipolysis, increasing the production of free fatty acids as a substrate for the production of body ketones.^14^

Ketoacidosis also occurs in pregnant women with diabetes in the presence of lower blood sugar, because pregnancy is associated with increased utilization by creating a hypermetabolic condition and reducing glucose production in the body.^15^

COVID‐19 virus has the ability to bind to the ACE2 receptor in the lungs and pancreas, and by inactivating it, it disrupts insulin secretion and hyperglycemia. Also, by increasing excessive fluid absorption through the RAAS system it can cause causes an irreversible state which can lead to underlying diseases occurrence such as diabetes mellitus, cardiovascular diseases, and thromboembolism.^16^

Some conditions such as the severity of lung involvement, arterial blood oxygenation rate and biochemical factors related to the severity of COVID‐19 infection in our patient, like other cases reported in pregnancy in the third trimester, are related to the severity of COVID‐19 infection. It seems that the metabolic acidosis of these patients is not consistent with the severity of COVID‐19,^2^, ^17^ and the association between COVID‐19 infection and pregnancy is sufficient to cause severe metabolic acidosis.

With regard to the severity of pulmonary involvement with COVID‐19 in this patient, it may be possible to suggest the patient's blood type, which was O positive, as a protective factor which is still a hypothesis.^18^ The patient was diagnosed with PTC in the first trimester of pregnancy. Regarding the association between the patient with COVID‐19 and PTC, although factors such as inflammation, immunity, obesity, and oxidative stress are involved, but it is not yet clear whether COVID‐19 infection could increase or decrease the risk of PTC. Besides, the presence of PTC as a baseline disease in our patient and its association with malignancies, is a COVID‐19 infection risk factor.^19^

Regarding the prognosis of COVID‐19 infection in PTC patients, studies so far have shown that in addition to age and underlying disease affecting on the hospitalization process of PTC patients with COVID‐19, the treatments and severity of COVID‐19 disease in PTC patients were not correlated. Overall, the rate of hospitalization and mortality in PTC patients are lower compared to other cancers at the time of COVID‐19 infection.^20^

## 5 CONCLUSION

Despite normal blood glucose levels, it is important to suspect ketoacidosis in a pregnant patient with acidosis. Urinary ketones should be monitored in any diabetic patient during periods of illness. Our case highlights the diagnostic and therapeutic challenges associated with euglycemic DKA and its associated complications. Due to sever acidosis association with fetal death, it is one of the mainstays of treatment, early detection and timely administration of fluids, carbohydrates, and insulin, to prevent hypoglycemia.

## AUTHOR CONTRIBUTIONS

A.F and F.M performed data entry and writing article. E.A and F.A patient management and data collection. F.M and A. F and B.KH performed data collection and article review.

## FUNDING INFORMATION

The Golestan University of Medical Sciences financially supported this work. The funder had no role in the design and conduction of the study; collection, management, analysis, and interpretation of the data; preparation, review, or approval of the manuscript; and decision to submit the manuscript for publication.

## CONFLICT OF INTEREST

The authors declare no competing interests.

## ETHICAL APPROVAL AND CONSENT TO PARTICIPATE

The study and all experimental protocols were approved by the Professional Ethics Committee of the Golestan University of Medical Sciences. An informed written consent was also obtained from the patient for the implementation of this project. All methods were carried out in accordance with relevant guidelines and regulations.

## CONSENT

Written informed consent was obtained from the patient to publish this report in accordance with the journal's patient consent policy.

## Data Availability

None.
